# Efficacy of MEK inhibition in a K-Ras-driven cholangiocarcinoma preclinical model

**DOI:** 10.1038/s41419-017-0183-4

**Published:** 2018-01-18

**Authors:** Mingjie Dong, Xianqiong Liu, Katja Evert, Kirsten Utpatel, Michele Peters, Shanshan Zhang, Zhong Xu, Li Che, Antonio Cigliano, Silvia Ribback, Frank Dombrowski, Antonio Cossu, John Gordan, Diego F. Calvisi, Matthias Evert, Yan Liu, Xin Chen

**Affiliations:** 1 Department of Gastroenterology, 307 Hospital of Academy of Military Medical Science, Beijing, China; 2 Department of Bioengineering and Therapeutic Sciences, University of California, San Francisco, CA USA; 3 Liver Center, University of California, San Francisco, CA USA; 4 School of Pharmacy, Hubei University of Chinese Medicine, Wuhan, Hubei China; 5 Institute of Pathology, University of Regensburg, Regensburg, Germany; 6 Institute of Pathology, University of Greifswald, Greifswald, Germany; 7 Tumor Immunology and Gene Therapy Center, Eastern Hepatobiliary Surgery Hospital, Second Military Medical University, Shanghai, China; 8 Unit of Pathology, Azienda Ospedaliero Universitaria Sassari, Sassari, Italy; 9 Department of Medicine, University of California, San Francisco, CA USA

## Abstract

Intrahepatic cholangiocarcinoma (iCCA) is a deadly malignancy with limited treatment options. Gain-of-function mutations in *K-Ras* is a very frequent alteration, occurring in ~15 to 25% of human iCCA patients. Here, we established a new iCCA model by expressing activated forms of Notch1 (NICD) and K-Ras (K-Ras^V12D^) in the mouse liver (K-Ras/NICD mice). Furthermore, we investigated the therapeutic potential of MEK inhibitors in vitro and in vivo using human CCA cell lines and K-Ras/NICD mice, respectively. Treatment with U0126, PD901, and Selumetinib MEK inhibitors triggered growth restraint in all CCA cell lines tested, with the most pronounced growth suppressive effects being observed in *K-Ras* mutant cells. Growth inhibition was due to reduction in proliferation and massive apoptosis. Furthermore, treatment of K-Ras/NICD tumor-bearing mice with PD901 resulted in stable disease. At the molecular level, PD901 efficiently inhibited ERK activation in K-Ras/NICD tumor cells, mainly leading to increased apoptosis. Altogether, our study demonstrates that K-Ras/NICD mice represent a novel and useful preclinical model to study K-Ras-driven iCCA development and the effectiveness of MEK inhibitors in counteracting this process. Our data support the usefulness of MEK inhibitors for the treatment of human iCCA.

## Introduction

Cholangiocarcinoma (CCA) is a type of malignancy with tumor cells arising within the liver or bile ducts with features of cholangiocyte differentiation^1,2^. In recent years, the incidence rate of CCA has been raising in the Western world^3,4^. Anatomically, CCA can be classified as intrahepatic (iCCA), perihilar (pCCA), and distal cholangiocarcinoma (dCCA). Hepatocellular carcinoma (HCC) and iCCA are the most common primary liver cancer, accounting for over 95% of all cases of primary liver cancer reported annually. iCCA is a deadly malignancy with limited treatment options. Surgical resection and liver transplantation are the only curative treatment approaches, but they can only be applied for early stage iCCA patients^1^. Unfortunately, most of iCCA cases are diagnosed at advanced stage, when curative treatments are not feasible. The combination of gemcitabine and cisplatin is the standard of care treatment for iCCA patients^5^. However, this therapeutic strategy has limited efficacy, with a median overall survival limited to 11.7 months^5^. As FDA-approved targeted therapies for iCCA are lacking, iCCA remains a deadly malignancy with a 5-year survival rate lower than 10%^6^.

Gain-of-function mutations of the *K-Ras* gene represent one of the most frequent alterations in iCCA. Indeed, multiple studies indicate that K-Ras mutations could be found in ~15–25% of human iCCAs^7–10^. Activated *K-Ras* mutations lead to constitutive hyper-activation of the Raf-MEK-ERK cascade (also known as the mitogen-activated protein kinase pathway or MAPK), an evolutionary conserved signaling pathway driving cell proliferation and survival. Targeting the oncogenic forms of K-Ras has been proven to be highly problematical. This depends on the fact that the K-Ras protein does not contain pockets or active sites that can be exploited for binding drugs. In addition, GTP and GDP bind extremely tightly to K-Ras, making it arduous to identify or design drugs that are effective competitive inhibitors^11^. Much effort has consequently been devoted to inhibit its downstream effectors, including Raf and MEK1/2 proteins^11^. In particular, MEK1/2 inhibitors have been extensively investigated in vitro, in preclinical models, and tested in clinical trials^12,13^. For instance, the MEK1/2 inhibitor Trametinib has been approved by the FDA for the treatment of *B-Raf* mutant metastatic and unresectable melanoma^14^. Despite the advances in the development of MEK inhibitors for cancer treatment, whether these drugs are useful for the treatment of iCCA, especially those with *K-Ras-*activating mutations, has been assessed only marginally, both in vitro and in vivo.

Here, we sought to determine the therapeutic potential of MEK inhibitors in a panel of human iCCA cell lines as well as in a novel mouse iCCA preclinical model characterized by the concomitant activation of *K-Ras*^*G12D*^ mutant allele and overexpression of an activated/cleaved form of Notch1 (NICD) (K-Ras/NICD). Our study suggests the efficacy of MEK inhibitors against K-Ras mutant iCCAs, supporting the further development of drugs targeting MEK1/2 for the treatment of *K-Ras* mutant iCCA.

## Results

### K-Ras mutant human CCA cell lines are highly sensitive to MEK inhibitors

As a first step to evaluate the therapeutic potential of MEK inhibitors for the treatment of iCCA, we collected seven human CCA cell lines. We sequenced the cell lines for *K-Ras* mutations and found that KKU213, HuCCT1, and RBE harbor activated *K-Ras* mutations, whereas the remaining four CCA cell lines, including KMCH, Huh28, MzCHa1, and OCUG, display wild-type *K-Ras* alleles (Supplemental Table 1). As surrogate marker of MAPK pathway activation, we assessed the levels of phosphorylated/activated (p)-ERK1/2 proteins in the seven cell lines. We found that p-ERK1/2 was expressed in all CCA cell lines irrespective of *K-Ras* mutation status (Supplemental Figure 1). Subsequently, we treated the seven cell lines with the MEK inhibitor U0126. U0126 is the most widely used and highly selective MEK1/2 inhibitor for in vitro studies^15^. We found that U0126 efficiently inhibits the growth of all CCA cell lines with IC_50_ ranging from ~20 to 100 μM (Supplemental Table 1). Of note, *K-Ras* mutant CCA cell lines resulted to be more sensitive than *K-Ras* wild-type CCA cells to U0126 administration (Supplemental Table 1).

**Fig. 1:**
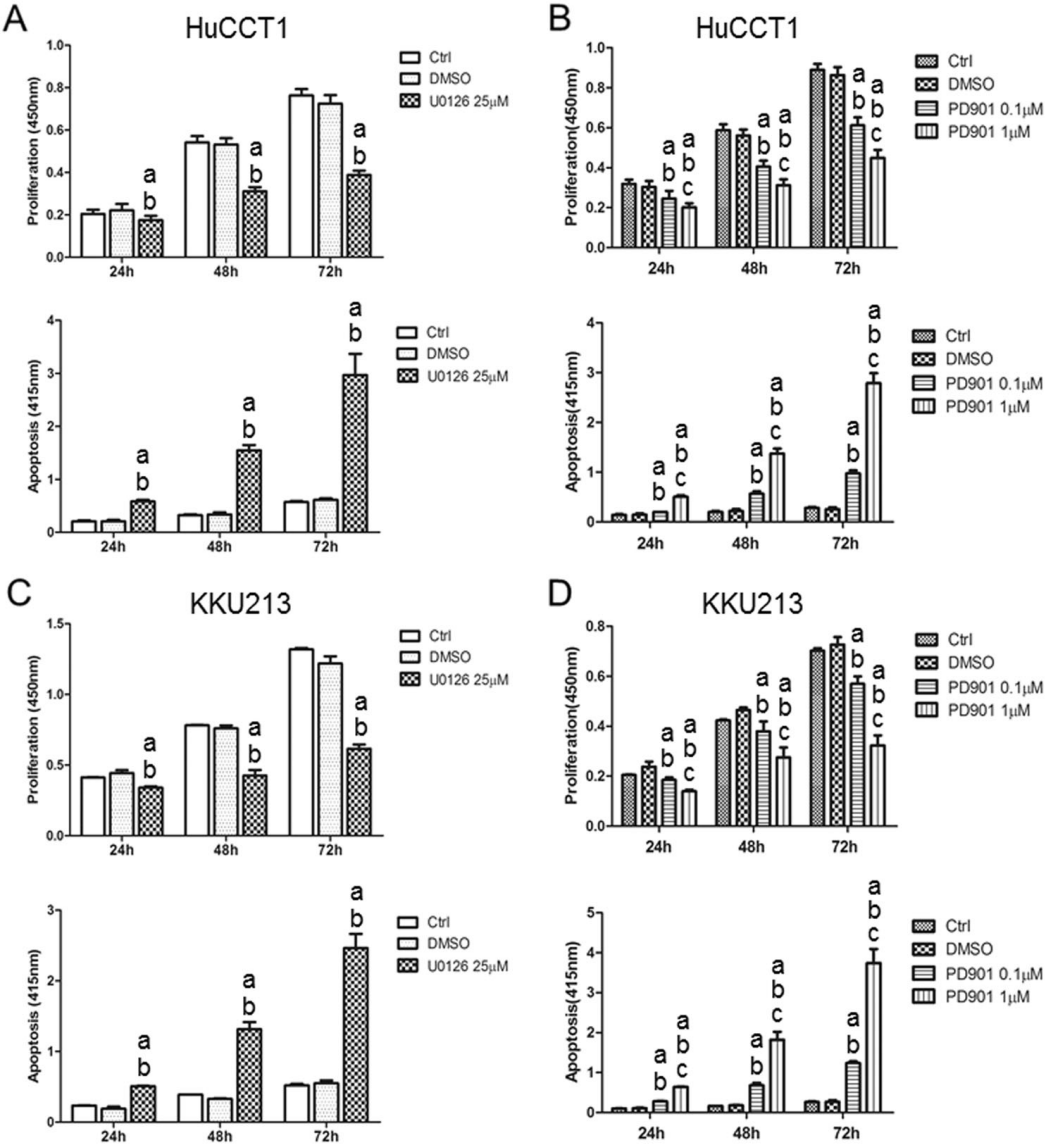
Effect of the MEK inhibitors U0126 and PD901 on the cell proliferation and apoptosis of *K-Ras* mutant HuCCT1 and KKU213 human CCA cell lines. **a**, **b** Cell proliferation and apoptosis rates of HuCCT1 cells treated with U0126 (**a**) or PD901 (**b**). **c**, **d** Cell proliferation and apoptosis rates of KKU213 cells treated with U0126 (**c**) or PD901 (**d**). Tukey Kramer test, *p* < 0.05, **a** vs. untreated cells; **b** vs. DMSO-treated cells; **c** vs. PD901 0.1 µM

Subsequently, we performed detailed analysis of MEK inhibitors on the growth of CCA cells by determining the proliferation and apoptosis rates in four selected CCA cell lines. For this purpose, we selected two *K-Ras* mutant (HuCCT1 and KKU213) and two *K-Ras* wild-type (KMCH and Huh28) CCA cell lines that were treated with U0126. Administration of U0126 induced a slight decrease in proliferation and a prominent increase in apoptosis in the four CCA cell lines tested (Fig. 1 and Supplemental Figure 2). Once again, the highest growth restraint was detected in *K-Ras* mutant cell lines (Fig. 1 and Supplemental Fig. 2).

**Fig. 2:**
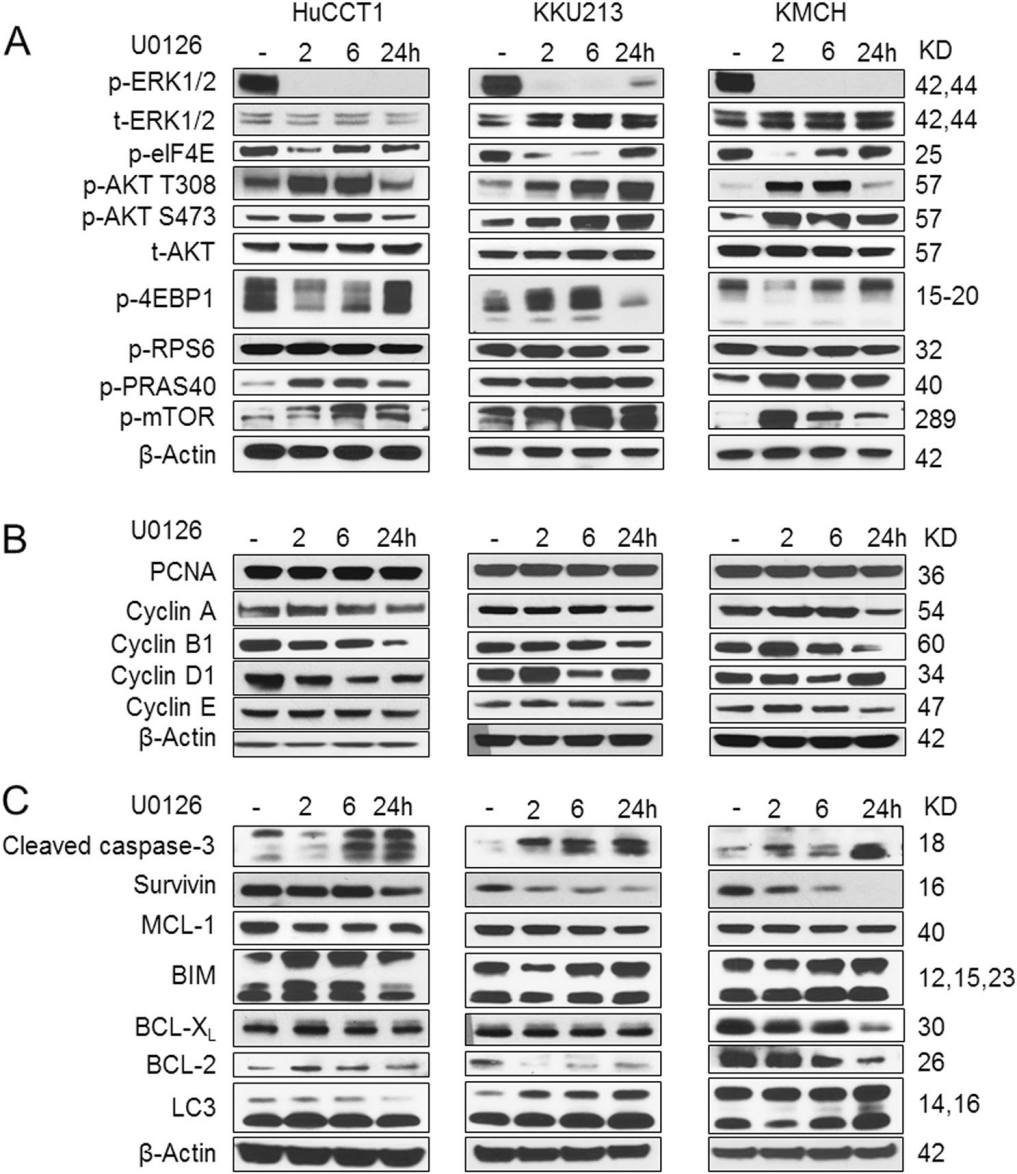
Biochemical analysis of U0126-treated human CCA cell lines. *K-Ras* mutant HuCCT1 and KKU213 cells as well as *K-Ras* wild-type KMCH cells were treated with U0126 at IC_50_ concentration, and analyzed at 0, 2, 6, and 24 h post treatment for **a** Ras/MAPK and AKT/mTOR pathways; **b** cell proliferation proteins; and **c** cell apoptosis-related proteins

At the biochemical level, U0126 efficiently inhibited p-ERK1/2 expression and its downstream target p-eIF4E (Fig. 2a). A feedback activation of AKT cascade, as indicated by phosphorylated/activated levels (p-) of AKT(S473), AKT(T308), and PRAS40, was noted in both *K-Ras* mutant and wild-type CCA tumor cells, especially at the early time points (Fig. 2a). However, no consistent upregulation of mTOR signaling, as revealed by phosphorylated/activated (p)-mTOR, (p)-RPS6, and (p)-4EBP1 expression was detected, suggesting that elevated levels of the AKT cascade do not always lead to upregulation of mTOR signaling in U0126-treated human CCA cell lines. As concerns proliferation proteins, levels of Cyclin A and Cyclin B1 were consistently downregulated in U0126-treated cells (Fig. 2b). Regarding apoptosis and autophagy pathways, expression of cleaved caspase-3 was increased, indicating that U0126 promotes apoptosis in human CCA cells. Furthermore, we found the consistent downregulation of pro-survival protein Survivin by U0126 in all CCA cells tested (Fig. 2c). The levels of other apoptosis-related proteins, including Mcl-1, Bim, BCL-XL, and BCL-2, did not change consistently (Fig. 2c).

While U0126 is widely used in vitro to study the efficacy of MEK inhibitor for cancer treatment, it is not suitable for in vivo experiments. PD0325901 (PD901) was developed to achieve high membrane permeability and high systematic exposure in vivo^16^ and it has been widely used for preclinical and clinical studies^12,13^. We therefore treated the four CCA cell lines with PD901 and investigated whether PD901 also regulates cell proliferation and apoptosis in human CCA cells. Indeed, similar to what we observed for U0126, a minor decline in proliferation and massive apoptosis was detected in all PD901-treated cell lines in a dose-dependent manner, especially in *K-Ras* mutant cells, when compared with solvent-treated and untreated cells (Fig. 1 and Supplemental Figure 2). In addition, we evaluated the effect of Selumetinib, a MEK inhibitor widely used in clinical trials (https://clinicaltrials.gov) on the same cell lines (Supplemental Figure 3). Once again, the highest growth restraint was achieved in K-Ras CCA mutant cells, although significant growth inhibition was also detected in K-Ras wild-type cells (Supplemental Figure 3). Equivalent results were also obtained, in a dose-dependent manner, when subjecting the four CCA cell lines to treatment with the ERK1/2 inhibitor SCH772984 (Supplemental Figure 4).

In summary, our in vitro data suggest that MEK (and ERK) inhibitors may be effective against human CCA cells, especially those with *K-Ras* mutations.

### Activated Notch1 synergizes with K-Ras^G12D^ to promote intrahepatic cholangiocarcinoma development in mice

**Fig. 3:**
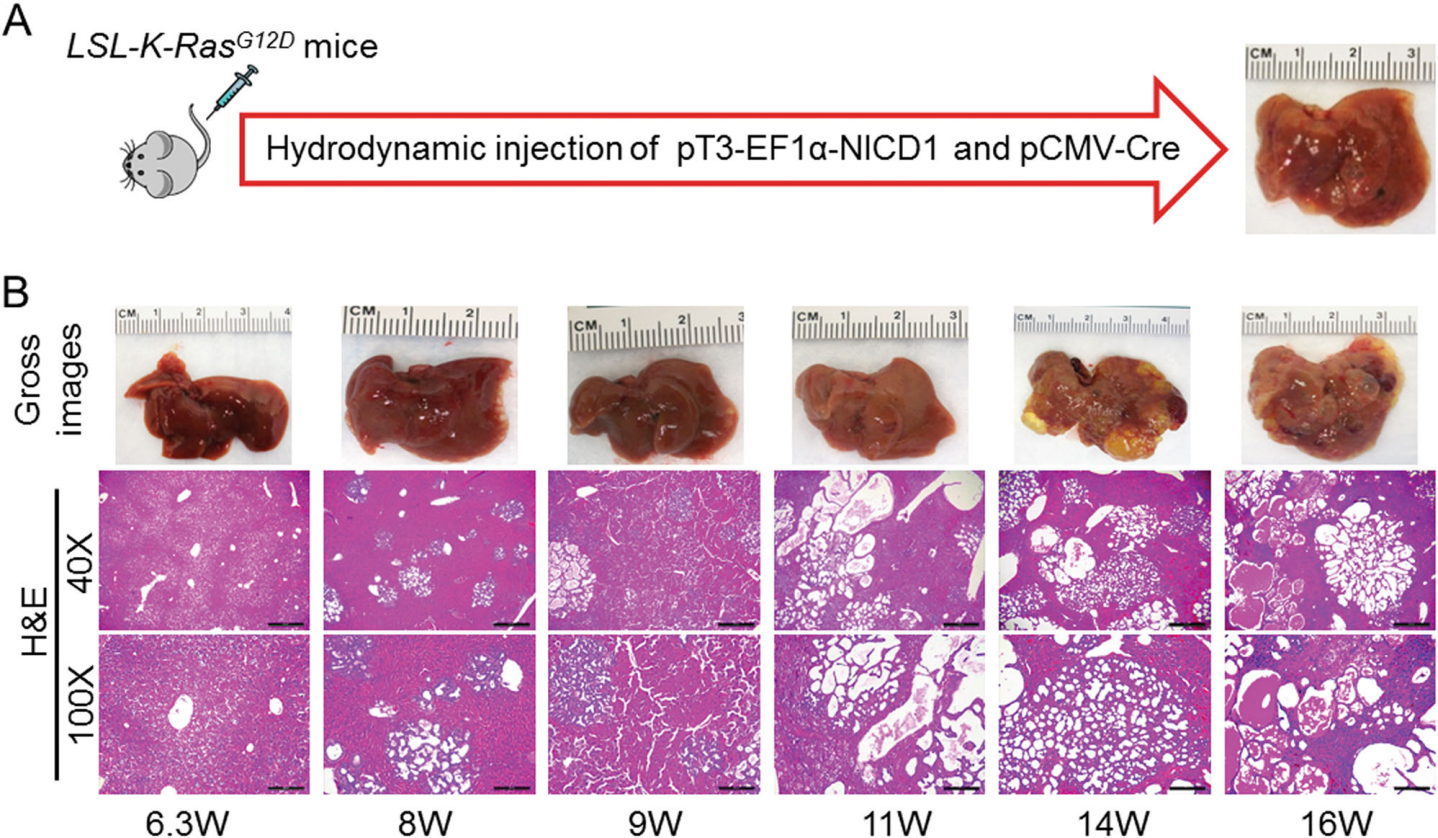
Activated Notch1 (NICD) synergizes with *K-Ras*^G12D^ to promote iCCA development in mice. **a** Study design. **b** Gross images and H&E staining of K-Ras/NICD mouse liver at various time points. W: weeks post injection. Scale bars: 500 μm in x40, 200 μm in x100

Next, to evaluate the importance of MEK inhibitors on iCCA growth in vivo, we developed a mouse model harboring a mutant, oncogenic form of *K-Ras* gene in the liver. Specifically, we hydrodynamically injected Cre recombinase into *LSL-K-Ras*^*G12D*^ mice (*n* = 5). Briefly, *LSL-K-Ras*^*G12D*^ mice carry a Lox-Stop-Lox (LSL) sequence followed by the *K-Ras*^*G12D*^ point mutation allele^17^. In these mice, Cre recombinase deletes the LSL cassette and allows the expression of mutant K-Ras, which is locked in a constitutively active, oncogenic conformation^17^. We found that over long term, no liver tumors developed in *LSL-K-Ras*^*G12D*^ mice (data not shown). Histological examination showed that the hepatic parenchyma of Cre-injected *LSL-K-Ras*^*G12D*^ mice was completely normal (not shown), suggesting that activation of *K-Ras* alone is unable to trigger carcinogenesis in the mouse liver. The results are equivalent to those obtained following the hydrodynamic injection of the *pT3-EF1a-K-Ras*^*G12D*^ construct in mice^18^. Activated Notch signaling is widely implicated in human CCA development and progression^19,20^. Previous data from our group indicate that overexpression of *NICD* (the intracellular domain of Notch1) alone leads to iCCA formation over long time in mice^21^. Thus, we hypothesized that activated *Notch1* may synergize with *K-Ras*^*G12D*^ mutant to promote iCCA formation. To substantiate this hypothesis, we hydrodynamically co-injected Cre with NICD into the liver of *LSL-K-Ras*^*G12D*^ mice (Fig. 3a), thus allowing co-expression of NICD with *K-Ras*^*G12D*^ mutant (K-Ras/NICD; *n* = 10). Of note, liver tumors developed as early as 8 weeks post injection in K-Ras/NICD mice (Fig. 3b). By ~14–16 weeks post injection, all mice (*n* = 5) developed high tumor burden and were required to be killed (Fig. 3b).

Histologically, all liver tumors exhibited a glandular phenotype, indicating that the combined oncogenic effect of K-Ras/NICD signaling results in the exclusive development of iCCA, but not HCC or HCC/iCCA mixed tumors, in mice (Fig. 3b). Some of the tumors consisted of small ductular structures with variable amount of desmoplastic stroma, while others exhibited a prominent cystic morphology (Fig. 3b). Some tumors showed both phenotypes intermingling with each other. Cellular atypia was low in cystic lesions, while the other tumors showed moderate and sometimes severe cytologic atypia, including an increase of mitotic figures and apoptotic bodies. An additional sign of malignancy was the invasion and destruction of the surrounding hepatocellular parenchyma.

**Fig. 4:**
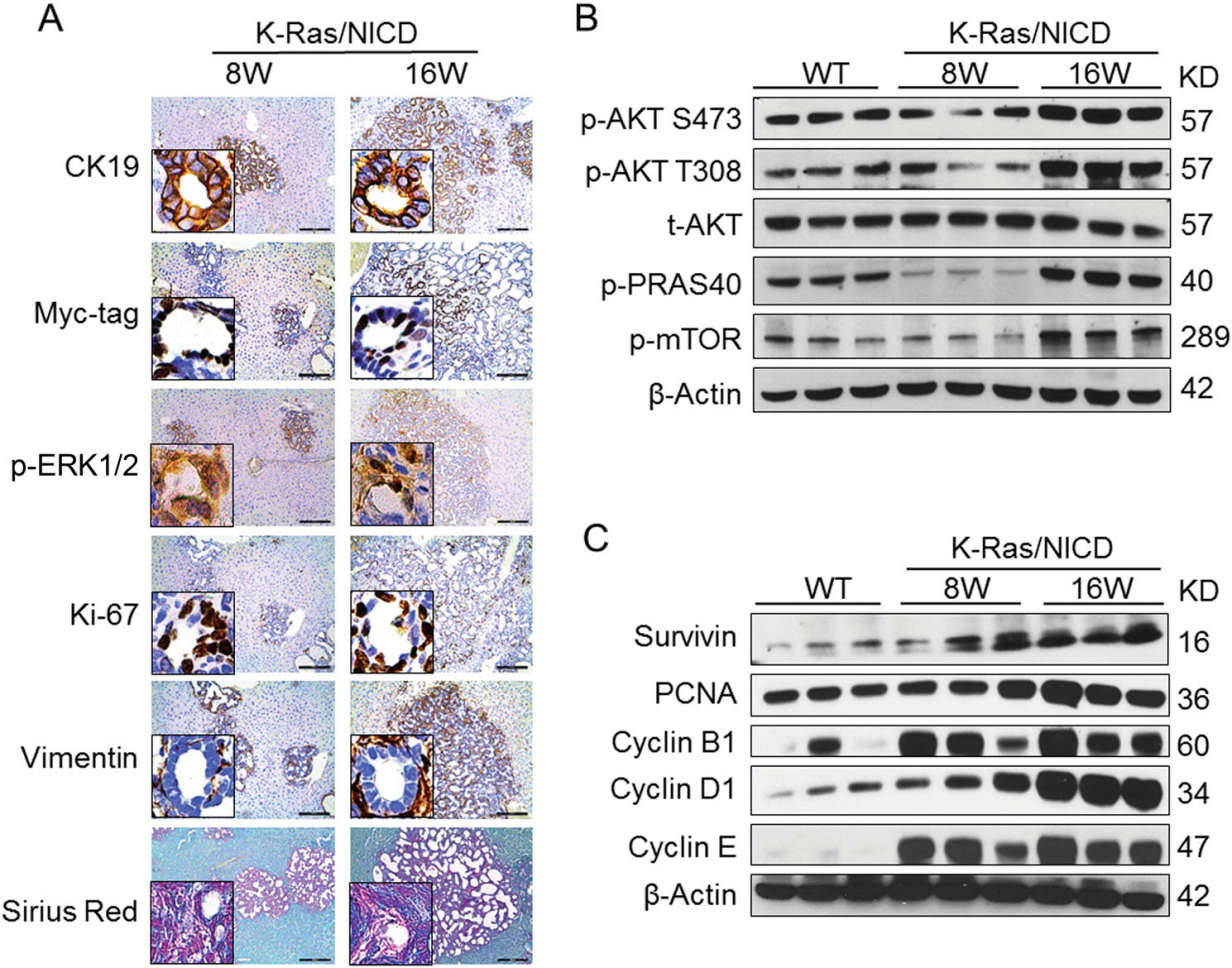
Molecular and biochemical features of K-Ras/NICD cholangiocellular tumors. **a** Immunohistochemical staining of K-Ras/NICD tumors at 8 weeks (8 W) and 16 weeks (16 W) post-hydrodynamic injection. **b** Western blotting of AKT/mTOR pathway genes in wild-type normal liver (WT) and K-Ras/NICD tumors at 8 W and 16 W post-hydrodynamic injection. **c** Western blotting of cell proliferation and apoptosis-related genes in WT normal liver and K-Ras/NICD tumors at 8 W and 16 W post-hydrodynamic injection

At the molecular level, all K-Ras/NICD tumor cells were positive for the biliary epithelial cell marker CK19, confirming that tumors were indeed iCCA (Fig. 4a). In addition, ectopically expressed *NICD* was visualized by Myc-tag immunostaining (Fig. 4a), while activation of *K-Ras*^*G12D*^ mutant was validated by elevated levels of one of its major downstream effector, namely p-ERK1/2 (Fig. 4a). K-Ras/NICD iCCA cells were highly proliferative as indicated by an increase in mitotic figures and Ki67-positive nuclear staining (Fig. 4a). Human iCCA is well known to exhibit an extensive desmoplasia^22^. Accordingly, K-Ras/NICD tumors displayed a high desmoplastic reaction, as revealed by histologic analysis and highlighted by immunoreactivity for vimentin (VIM) in the stromal fibroblasts and myofibroblasts as well as strong Sirius Red staining of the collagen fibers within the tumor tissue (Fig. 4a). At the biochemical level, the AKT/mTOR pathway, a major signaling cascade promoting cell survival, was found to be activated in late-stage K-Ras/NICD tumors (Fig. 4b). Cell proliferation-related proteins, including PCNA, Cyclin B1, Cyclin D1, and Cyclin E, were also highly expressed in K-Ras/NICD iCCA. Similarly, Survivin, an important anti-apoptosis protein, was upregulated in these tumors (Fig. 4c).

In summary, our study demonstrates that activated Notch1 synergizes with *K-Ras*^*G12D*^ mutant to promote development of cholangiocellular tumors in mice that closely resemble human iCCA. Thus, K-Ras/NICD mice represent a novel murine model to study K-Ras-driven iCCA development in vivo.

### Treatment with the MEK inhibitor PD901 leads to stable disease in K-Ras/NICD mice

**Fig. 5:**
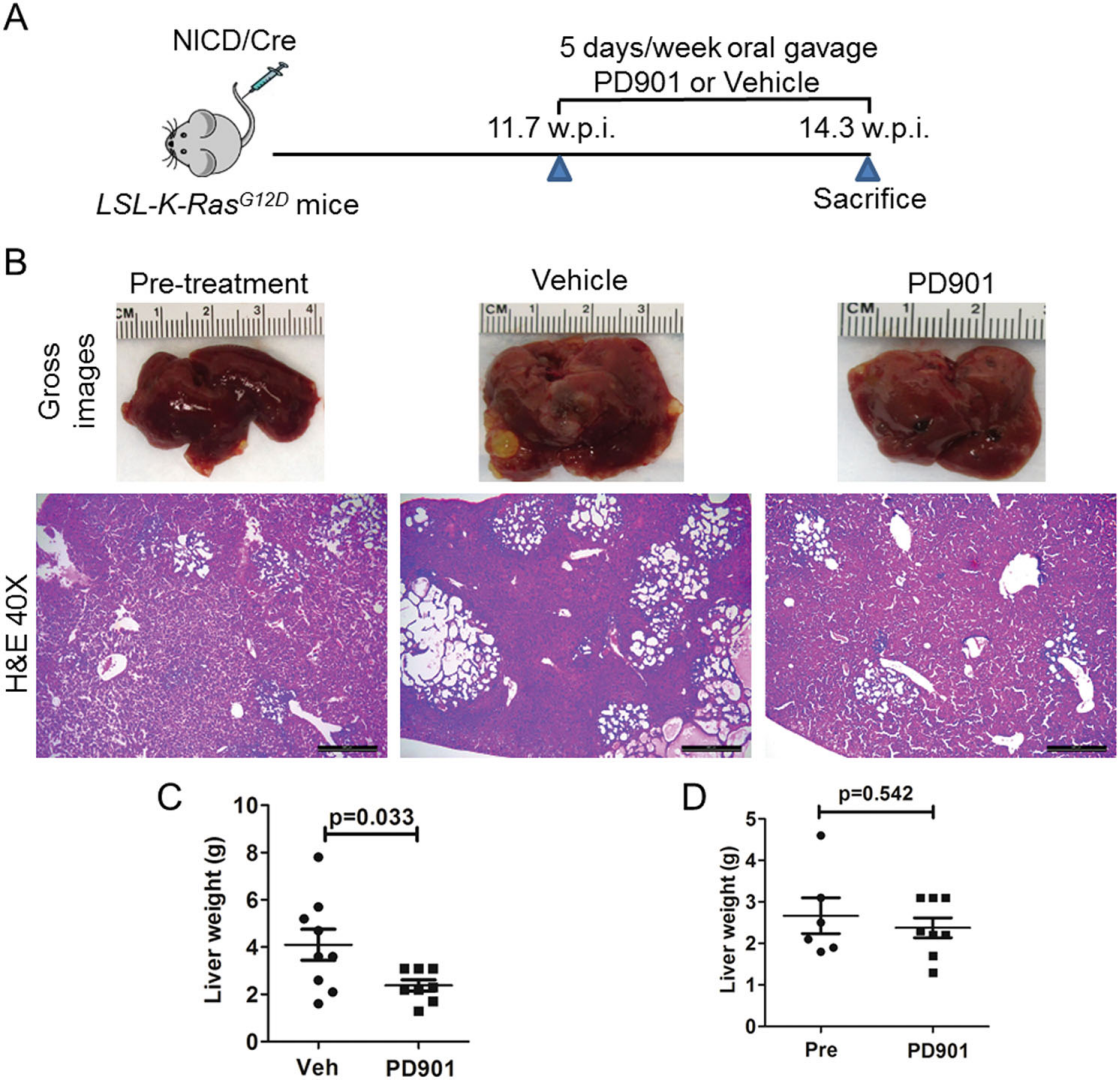
Treatment with the MEK inhibitor PD901 leads to stable disease in K-Ras/NICD mice. **a** Study design. **b** Gross images and H&E staining of K-Ras/NICD mouse liver at 11.7 weeks post injection (pre-treatment); vehicle treated at 14.3 weeks post injection as well as PD901 treated at 14.3 weeks post injection. Scale bars: 500 μm; **c** Liver weight comparison in vehicle and PD901-treated K-Ras/NICD mice; **d** Liver weight comparison in pretreated and PD901-treated K-Ras/NICD mice. Pre Pre-treatment, Veh Vehicle

Having established a *K-Ras*-driven iCCA model, we next investigated the potential therapeutic activity of MEK inhibitors in this iCCA preclinical model. For this purpose, we hydrodynamically transfected Cre and NICD into *LSL-K-Ras*^*G12D*^ mice. At 11.7 weeks post injection, when K-Ras/NICD mice display low to moderate iCCA tumor burden, a cohort of mice (*n* = 6) was harvested as pre-treatment baseline measurement. In the meantime, we started to treat K-Ras/NICD mice with either vehicle (*n* = 9) or the MEK inhibitor PD901 (*n* = 8). PD901 was selected for the in vivo treatment as it has been already evaluated in experimental models^23–25^ and is currently investigated in clinical trials of multiple tumor types (Clinical trial number: NCT02510001, NCT02022982, and NCT02039336). All mice were killed at 14.3 weeks post injection (Fig. 5a). We used total liver weight as the measurement of iCCA tumor burden in mice^26^. Of note, PD901-treated mice displayed a significantly lower total liver weight than vehicle-treated mice (Fig. 5b, 5c). In addition, PD901-treated mice had a similar tumor burden to that of the pretreated mouse cohort (Fig. 5d), suggesting that PD901 led to stable disease in K-Ras/NICD mice.

**Fig. 6:**
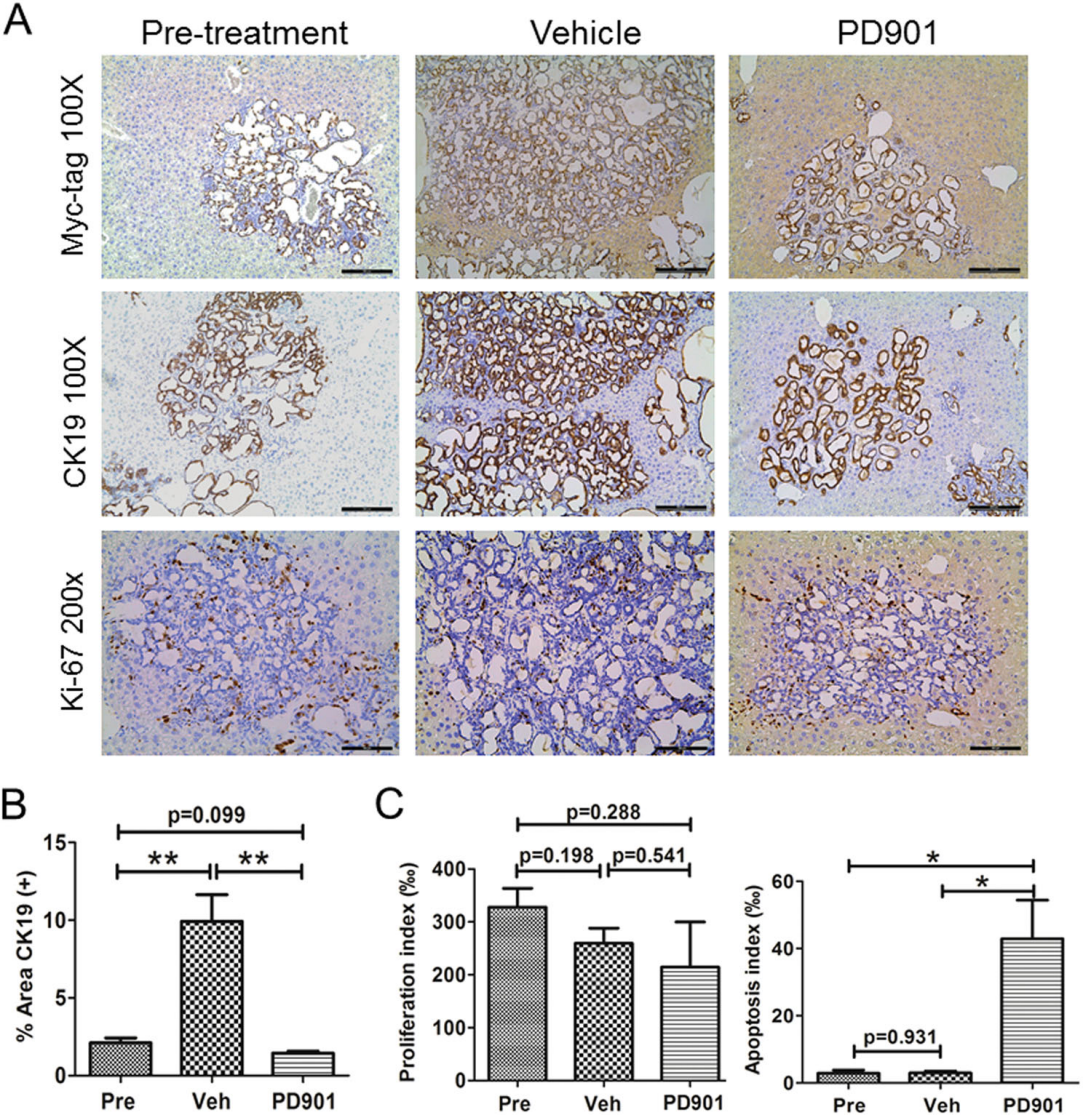
PD901 treatment triggers apoptosis in K-Ras/NICD mouse lesions. **a** Immunohistochemical staining of pretreated, vehicle-treated, and PD901-treated K-Ras/NICD mice; **b** Quantification of CK19(+) area as a measurement of tumor burden in three mouse cohorts. **c** Proliferation index and apoptosis index. Scale bars: 200 μm for Myc-tag and CK19 staining; 100 μm in Ki67 staining. Student’s *t* test, *p < 0.05; **p < 0.01. Pre Pre-treatment, Veh Vehicle

**Fig. 7:**
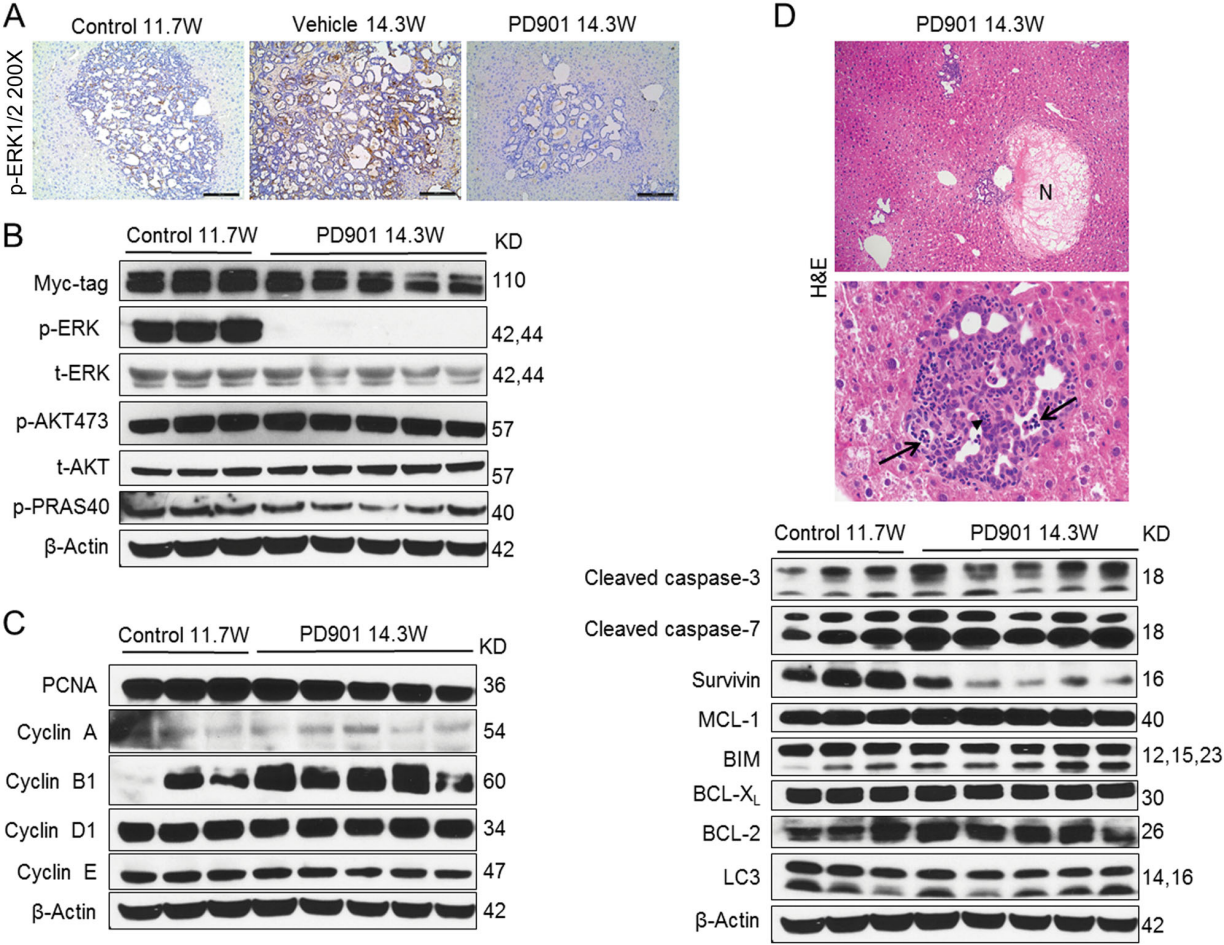
Biochemical analysis of PD901-treated K-Ras/NICD mice. **a** Immunohistochemical staining of p-ERK in pretreated, vehicle-treated, and PD901-treated K-Ras/NICD mice; **b** Western blotting analysis of MAPK and AKT pathways in pretreated and PD901-treated K-Ras/NICD mice; **c** Western blotting analysis of cell proliferation proteins; **d** histopathological analysis of PD901-treated K-Ras/NICD livers (upper and middle panel) and western blot analysis of apoptosis-related proteins (lower panel). Treatment with PD901 resulted in complete tumor necrosis (upper panel; indicated as N) as well as by infiltration of neoplastic lesions by inflammatory cells (middle panel; arrowhead indicates granulocytes) and induction of apoptosis (middle panel; apoptotic debris are indicated by arrows)

Histologically, we found that K-Ras/NICD tumors in pretreated, vehicle-treated, and PD901-treated mice were highly similar. Indeed, treatment with PD901 did not alter the histomorphologic features of the lesions and all tumors were still either cystic, ductular, or combined iCCA (Fig. 5b). Also, all K-Ras/NICD tumor cells were CK19 positive (Fig. 6a) and expressed ectopically injected Myc-tagged NICD following PD901 administration (Fig. 6a). As another measurement of tumor burden, we quantified the CK19(+) area in pretreated, vehicle-treated, and PD901-treated mouse liver samples. We found that the CK19(+) area was larger in vehicle-treated liver samples than that in pretreated ones. PD901 significantly decreased CK19(+) area when it was compared with vehicle-treated liver tissues; and PD901-treated and pre-treatment liver specimens showed a similar CK19(+) area (Fig. 6b). In line with the decrease in tumor burden in PD901-treated animals when compared to vehicle-treated ones, we found histopathologic signs of tumor regression, such as accumulation of apoptotic bodies and fibrinoid or hemorrhagic necrosis, often accompanied by an inflammatory reaction in some of the tumors (Fig. 7d). Subsequently, proliferation and apoptosis rates were determined in iCCA lesions. Of note, PD901 treatment did not significantly decreased iCCA cell proliferation but, in contrast, strongly induced tumor cell apoptosis (Fig. 6c). The increased apoptosis was confirmed by the conspicuous accumulation of apoptotic cells that was already noticed in conventional histopathology (Fig. 7d).

Next, we investigated the signaling pathways that might be regulated by PD901 in K-Ras/NICD iCCA lesions. We found that tumor burden was higher in vehicle-treated than in PD901-treated tumor samples (Figs. 5c and 6b). Thus, to exclude that the observed differences were due to the different tumor burden, we compared PD901-treated K-Ras/NICD liver lysates with pretreated K-Ras/NICD counterparts (i.e., K-Ras/NICD iCCA at 11.7 weeks post injection) that display an equivalent liver load by iCCA lesions (Figs. 5d and 6b). As expected, western blotting demonstrated similar Myc-tag levels (indicating the hydrodynamically injected NICD) in the pre-treatment and PD901-treated livers (Fig. 7b). As a biomarker of PD901 efficacy, we found that p-ERK1/2 expression was completely abolished in PD901-treated K-Ras/NICD livers (Fig. 7a). No further activation of AKT was observed after treatment with PD901 in mice (Fig. 7b). The expression of proteins involved in cell proliferation, including PCNA, Cyclin A, Cyclin B1, Cyclin D1, and Cyclin E, was not altered (Fig. 7c). As concerns apoptosis-related proteins, a mild but consistent increased expression of cleaved caspase-3 and -7 was detected in PD901-treated livers (Fig. 7d). Expression of Mcl-1, Bim, BCL-XL, and BCL-2 did not differ between control and PD901-treated livers (Fig. 7d). However, a consistent downregulation of Survivin expression in PD901-treated tumors was observed (Fig. 7d).

In summary, our study indicates that targeting MEK proteins in K-Ras/NICD mice leads to stable disease mainly due to the induction of iCCA cell apoptosis.

### Frequent activation of the MAPK pathway in human cholangiocarcinoma

**Fig. 8:**
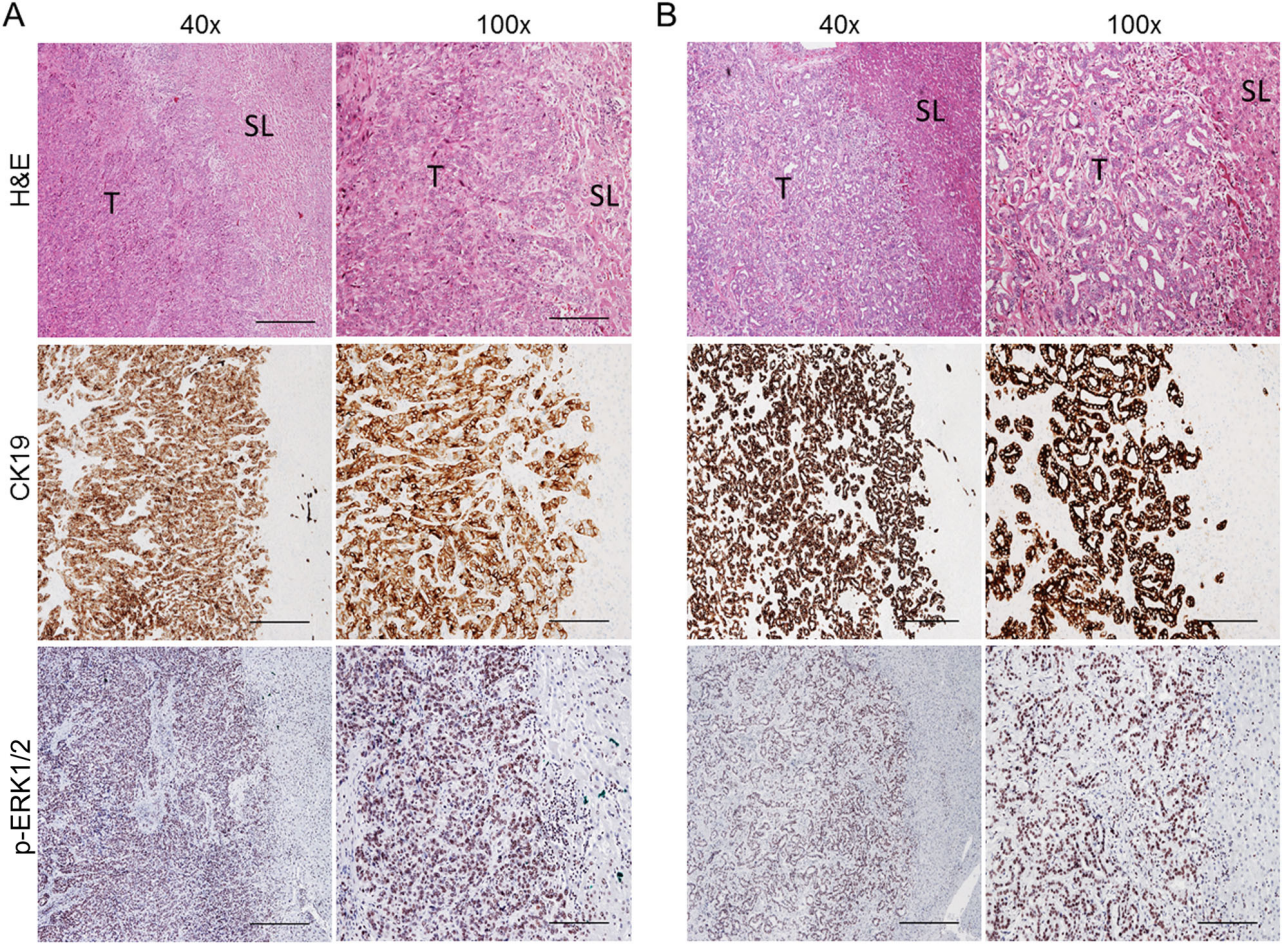
Ubiquitous activation of the MAPK pathway in human intrahepatic cholangiocarcinoma (iCCA) with or without K-Ras mutations. Immunohistochemical pattern of phosphorylated/activated (p)-ERK1/2, a surrogate marker of MAPK pathway activation, in two human iCCA specimens, exhibiting mutant *K-Ras*^*G12D*^ allele (**a**) and wild-type *K-Ras* allele (**b**), respectively. The two iCCA samples are depicted in two magnifications (x40 and x100) and show strong nuclear immunoreactivity for p-ERK1/2 in the tumor part (T) when compared with non-tumorous surrounding liver tissues (ST). H&E hematoxylin and eosin staining. Scale bar: 500 μm in x40, 200 μm in x100

Finally, to stratify the iCCA patients who might benefit from the use of MEK inhibitors, we assessed the mutations frequency in K-Ras as well as the activation of the MAPK signaling in a collection of human iCCA specimens. Mutations in *K-Ras* were detected in 20 of 98 (20.4%) samples. All specimens harboring mutations in *K-Ras* displayed activation of the Ras/MAPK signaling, as assessed by immunohistochemistry for p-ERK1/2 proteins (Fig. 8a). Of note, activation of the Ras/MAPK pathway by immunohistochemistry was detected in additional 53 iCCA samples with wild-type *K-Ras* from the same collection (Fig. 8b), indicating the almost ubiquitous induction of this signaling cascade in human iCCA.

## Discussion

Preclinical murine models are critical to study the oncogenic potential of a given gene as well as for testing the therapeutic potential of novel therapies for cancer treatment. Because of the high prevalence of *K-Ras* mutations in human iCCA, several mouse models with activating *K-Ras* mutations have been developed. For instance, it has been reported that *K-Ras*^*G12D*^ synergizes with loss of p53 to promote iCCA formation by ~18 weeks of age in mice^27^. However, in this model, HCC or mixed HCC/iCCA could also develop^27^. In another model, using inducible Cre recombinase, Marsh et al. showed that *AhCreER2*^*T+*^*;Pten*^*f/f*^*;K-Ras*^*G12D*^ mice developed iCCA ~43 days after Cre induction by β-naphthoflavone administration^28^. Although these studies support a pivotal role of *K-Ras* mutation in cholangiocarcinogenesis, none of these animal models has been used to characterize the therapeutic potential of drugs for iCCA treatment. In our current study, we reported the establishment of a novel murine iCCA model induced by activated *K-Ras* and *Notch1* alleles. Consistent with previous studies^27,28^, activation of *K-Ras* alone in the mouse liver did not lead to any abnormality. Overexpression of NICD alone only induces iCCA formation after a long latency^29^. In contrast, concomitant activation of K-Ras and Notch1 significantly decreased the latency of tumor development, leading to iCCA formation by ~11 weeks post-hydrodynamic transfection. At the biochemical level, we demonstrated that K-Ras/NICD iCCAs display elevated levels of p-ERK1/2, a major signaling event downstream of the *K-Ras* oncogene. Importantly, iCCA development in this model is highly reproducible with 100% of mice developing iCCA by 14–16 weeks post-hydrodynamic transfection. In addition, no HCC or mixed HCC/iCCA lesions occur in K-Ras/NICD mice. Thus, the K-Ras/NICD mouse model is an ideal preclinical tool to evaluate the therapeutic potential of drugs targeting the Ras pathway for iCCA treatment.

The Raf-MEK-ERK cascade has been considered the major signaling event downstream of K-Ras. Once activated, it is critical for the regulation of cell growth, survival, and differentiation. Effects of directly targeting K-Ras have been largely unsuccessful to date^11^. In contrast, targeting signaling molecules downstream of K-Ras has led to the development of multiple small molecules with anticancer efficacy. Among all downstream molecules of K-Ras, the MEK1/2 proteins have a unique structure allowing the design of ATP-noncompetitive inhibitors that can lock MEK1/2 into an inactive form. In 1995, PD98059 was the first reported synthetic small molecule inhibitor of MEK1/2^30^. Since then, multiple MEK1/2 inhibitors have been developed^12,13^. PD901 is a highly selective allosteric MEK1/2 inhibitor with IC_50_ of 1 nM against activated MEK1/2^16^. It has been used to study the therapeutic efficacy of inhibiting MEK proteins in multiple preclinical studies^16^. PD901 has shown efficacy in preclinical models of Ras or Raf mutant acute myeloid leukemia^23^, colon cancer^24^, and pancreatic cancer^25^. However, its efficacy in iCCA has not been previously tested. Here, we demonstrate that the treatment of K-Ras/NICD mice with PD901 leads to stabilized disease mainly by promoting apoptosis in tumor cells. The results support the further testing of MEK1/2 inhibitors for the treatment of *K-Ras* mutant iCCAs in human patients. It is important to note that PD901 treatment in K-Ras/NICD did not lead to a significant decrease in cell proliferation (Fig. 6c), which may limit its therapeutic efficacy. Indeed, we observed that PD901 treatment resulted in stable disease but not tumor regression in K-Ras/NICD mice. This finding suggests that PD901 should be combined with small inhibitors or conventional chemotherapeutic agents possessing anti-proliferative activity for the treatment of iCCA. In particular, it is worth to note that while PD901 efficiently inhibited the Ras/MAPK pathway, the AKT/mTOR cascade remained elevated in K-Ras/NICD tumor tissues (Fig. 7b). Thus, it is likely that the activated AKT/mTOR pathway might be one of the driving forces for the sustained cell proliferation in PD901-treated K-Ras/NICD lesions as well in human iCCA, and requires to be further investigated. Therefore, the combination of PD901 with AKT/mTOR inhibitors might be helpful to induce growth restraint and tumor regression in K-Ras/NICD mice and, possibly, human iCCA.

Finally, the data obtained in CCA cell lines unraveled an anti-growth effect by MEK inhibitors even in *K-Ras* wild-type cells, thus suggesting the usefulness of these drugs in iCCA patients not carrying *K-Ras* mutation as well. This finding is important to be mentioned in light of the almost ubiquitous activation of ERK in iCCA patients that we have detected in the present study. This evidence is in accordance with data obtained in other tumor types, where the MEK/ERK pathway was found to be universally and robustly activated even in the absence of *Ras* mutations. In HCC, for instance, multiple genetic and epigenetic events disrupting the function of cellular suppressor of Ras, Raf, MEK, and ERK proteins were identified, resulting in unrestrained activity of the MEK/ERK cascade in the presence of wild-type Ras^31^. Additional investigation to evaluate the frequency and the importance of these alternative mechanisms of MEK/ERK activation in human iCCA is necessary. Furthermore, preclinical models of iCCA exhibiting activation of the MAPK pathway in the absence of *K-Ras* mutations should be established and subjected to MEK inhibitors in order to evaluate their relevance in vivo.

## Materials and methods

### Constructs and reagents

The constructs used for mouse injection, including Myc-tagged pT3-EF1α-NICD and pCMV/sleeping beauty transposase (pCMV/SB) plasmids, were described previously^21^. pCMV-Cre was obtained from Addgene (plasmid #11916). Plasmids were purified using the Endotoxin Free Maxi Prep Kit (Sigma-Aldrich, St. Louis, MO) before being injected into the mice. U0126 and PD0325901 (PD901) were purchased from LC Laboratories (Woburn, MA). Selumetinib and SCH772984 were purchased from Selleck Chemicals (Houston, TX).

### Hydrodynamic tail vein injection and mouse treatment

*LSL-K-Ras*^*G12D*^ mice in FVB/N background were kindly provided to us by Dr Allan Balmain of UCSF^17^. Hydrodynamic injections were performed as described previously^32^. Briefly, 20 μg of the plasmids encoding pT3-EF1α-NICD and pCMV-Cre along with sleeping beauty transposase (pCMV/SB) at a ratio of 25:1 were diluted in 2 ml saline (0.9% NaCl) for each mouse. Saline solution was filtered through a 0.22 μm filter and injected into the lateral tail vein of 6- to 8-week-old *LSL-K-Ras*^*G12D*^ mice within 7 s. PD901 was dissolved in 0.5% (w/v) hydroxy-propyl-methylcellulose (HPMT; Sigma) in water plus 0.2% v/v Tween 80 to a stock concentration of 3.33 mg/ml. PD901 (10 mg/kg/day) or vehicle was orally administered via gavage for 5 days per week. Mice were housed, fed, and monitored in accord with protocols approved by the committee for animal research at the University of California San Francisco (San Francisco, CA).

### Histology and immunohistochemistry

Mouse and human liver samples were fixed overnight in zinc formalin (Anatech Ltd.), embedded in paraffin, cut into 5-μm-thick sections, and placed on glass slides. Liver lesions were evaluated by three board-certified pathologists (M.E., K.E., and K.U.). Primary antibodies extensively validated by the manufacturers for immunohistochemistry were selected and listed in Supplemental Table 2. Briefly, slides were deparaffinized in xylene, rehydrated through a graded alcohol series and rinsed in PBS. After boiling in 0.01 M citrate buffer (pH 6.0) for 10 min in a microwave oven, the slides were cooled down at room temperature, then blocked with 5% goat serum and Avidin-Biotin Blocking Kit (Vector Laboratories, Burlingame, CA). Subsequently, slides were incubated with primary antibodies overnight at 4 °C. Slides were then subjected to 3% hydrogen peroxide for 10 min to quench endogenous peroxidase activity and subsequently the biotin-conjugated secondary antibody was applied at a 1:500 dilution for 30 min at room temperature. Finally, signal was visualized using the Vectastain ABC Elite Kit (Vector Laboratories In, Burlingame, CA) and developed with 3,3′-diaminobenzidine. Sections were counterstained with hematoxylin (Sigma). Negative controls were performed with the same procedure, and PBS was incubated as a substitute for the primary antibodies.

### Assessment of proliferation and apoptosis indices

Proliferation and apoptosis indices were determined in mouse tumor lesions by counting Ki67 and TUNEL-positive cells, respectively, on at least 3000 mouse tumor cells per sample. Indices are expressed as percentage of positive over total cells counted. TUNEL staining was conducted using the ApopTag^®^ Peroxidase In Situ Apoptosis Detection Kit (EMD Millipore, Burlington, MA), following the manufacturer’s protocol.

### Protein extraction and western blotting

Frozen mouse liver specimens and cultured cell samples were homogenized in Mammalian Protein Extraction Reagent (Thermo Scientific, Waltham, MA) containing the Complete Protease Inhibitor Cocktail and sonicated. Protein concentrations were determined with the Bio-Rad Protein Assay Kit (Bio-Rad, Hercules, CA) using bovine serum albumin as standard. Aliquots of 40 μg lysate were denatured by boiling in Tris-Glycine SDS Sample Buffer (Invitrogen), separated by SDS-PAGE, and transferred onto nitrocellulose membranes (Invitrogen, Grand Island, NY). Membranes were blocked in 5% non-fat dry milk in Tris-buffered saline containing 0.1% Tween 20 for 1 h and probed with specific antibodies listed in Supplemental Table 2. Each primary antibody was followed by incubation with horseradish peroxidase-secondary antibody diluted 1:10,000 for 1 h and then revealed with the SuperSignal West Pico Chemiluminescent Substrate (Pierce Chemical Co., New York, NY). Equal loading was assessed by Ponceau Red reversible staining as well as GAPDH and β-actin western blotting.

### In vitro experiments

Human CCA cell lines, including KKU213, HuCCT1, RBE, KMCH, Huh28, MzCHa1, and OCUG, (Supplemental table 4) were used for the in vitro studies. Cell lines were maintained as monolayer cultures in Dulbecco’s modified Eagle medium supplemented with 10% fetal bovine serum (FBS, Gibco, Grand Island, NY, USA) and 100 U/ml penicillin, 100 g/ml streptomycin (Gibco). For IC_50_ determination, cells were seeded in 24-well plates and treated with increasing doses of U0126 in triplicate for 48 h. Cells were stained with crystal violet. After washing, stained cells were treated with lysis solution and shaken gently on a rocking shaker for 20–30 min. Diluted lysate solutions were added to 96-well plates and OD was measured at 590 nm with a BioTek ELx808 Absorbance Microplate Reader. All cell line experiments were repeated at least three times in triplicate. Cell proliferation and apoptosis were assessed using the BrdU Cell Proliferation Assay Kit (Cell Signaling Technology Inc) and the Cell Death Detection Elisa Plus Kit (Roche Molecular Biochemicals, Indianapolis, IN), respectively, following the manufacturer's instructions.

### Human tissue samples

A collection of formalin-fixed, paraffin-embedded iCCA (*n* = 98) samples was used in the present study. The clinicopathological features of liver cancer patients are summarized in Supplemental Table 3. iCCA specimens were collected at the Medical Universities of Greifswald (Greifswald, Germany) and Sassari (Sassari, Italy). Institutional Review Board approval was obtained at the local Ethical Committee of the Medical Universities of Greifswald and Sassari. Informed consent was obtained from all subjects.

### K-Ras sequencing analysis

PCR was performed with 50 ng DNA on the FlexCycler (Analytik Jena, Jena, Germany) thermal cycler. Briefly, the reaction was carried out using the innuTaq HOT-A DNA Polymerase with 10× PCR buffer with KCl (Analytik Jena), in a total volume of 25 µl, containing 0.2 µm of each primer, 200 µm of each dNTP, 1.5 mM MgCl_2_, and 0.5 U DNA polymerase. An initial denaturation for 3 min at 94 °C was followed by 40 cycles of 94 °C for 45 s, 65 °C for 45 s, and 72 °C for 45 s, with a final elongation step of 10 min for 72 °C. Primers were obtained from Biomers (Ulm, Germany) and sequences were taken from the literature^28^. Sequencing was performed with the GenomeLab DTCS Quick Start Kit (Beckman Coulter, Pasadena, CA) on the GenomeLab GeXP (Beckman Coulter).

### Statistical analysis

Data analysis was performed with Prism 6 (GraphPad, San Diego, CA). All data are presented as Means ±SD. Comparisons were performed with Student’s two-tailed unpaired *t* test or Tukey Kramer test. *P* values <0.05 were considered statistically significant.

## Electronic supplementary material

Supplemental Figure 1 2 3 and 4

Supplemental Table 1 2 3 and 4
