# Supplementary material for: Efficacy of MEK inhibition in a K-Ras-driven cholangiocarcinoma preclinical model
Source: Cell Death Dis. 2018 Jan 18;9(2):31. doi: 10.1038/s41419-017-0183-4 (PMC5833851; doi:10.1038/s41419-017-0183-4)
Supplement: Supplementary file 1 — Supplemental Figure 1 2 3 and 4 [file 41419_2017_183_MOESM1_ESM.pptx]

## Slide 1
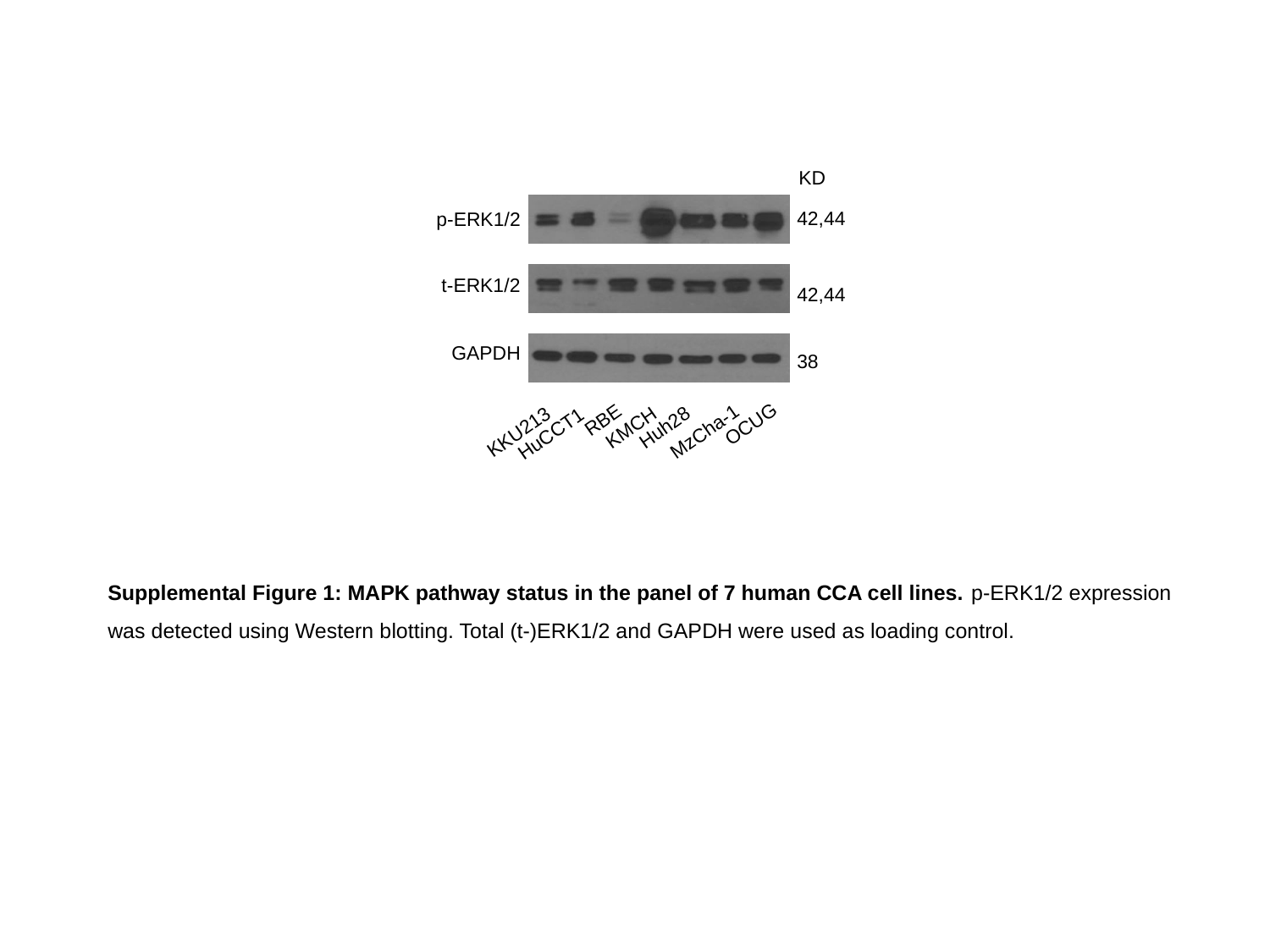

KD
42,44
p-ERK1/2
t-ERK1/2
42,44
GAPDH
38
OCUG
RBE
MzCha-1
Huh28
KKU213
KMCH
HuCCT1
Supplemental Figure 1: MAPK pathway status in the panel of 7 human CCA cell lines. p-ERK1/2 expression was detected using Western blotting. Total (t-)ERK1/2 and GAPDH were used as loading control.

## Slide 2
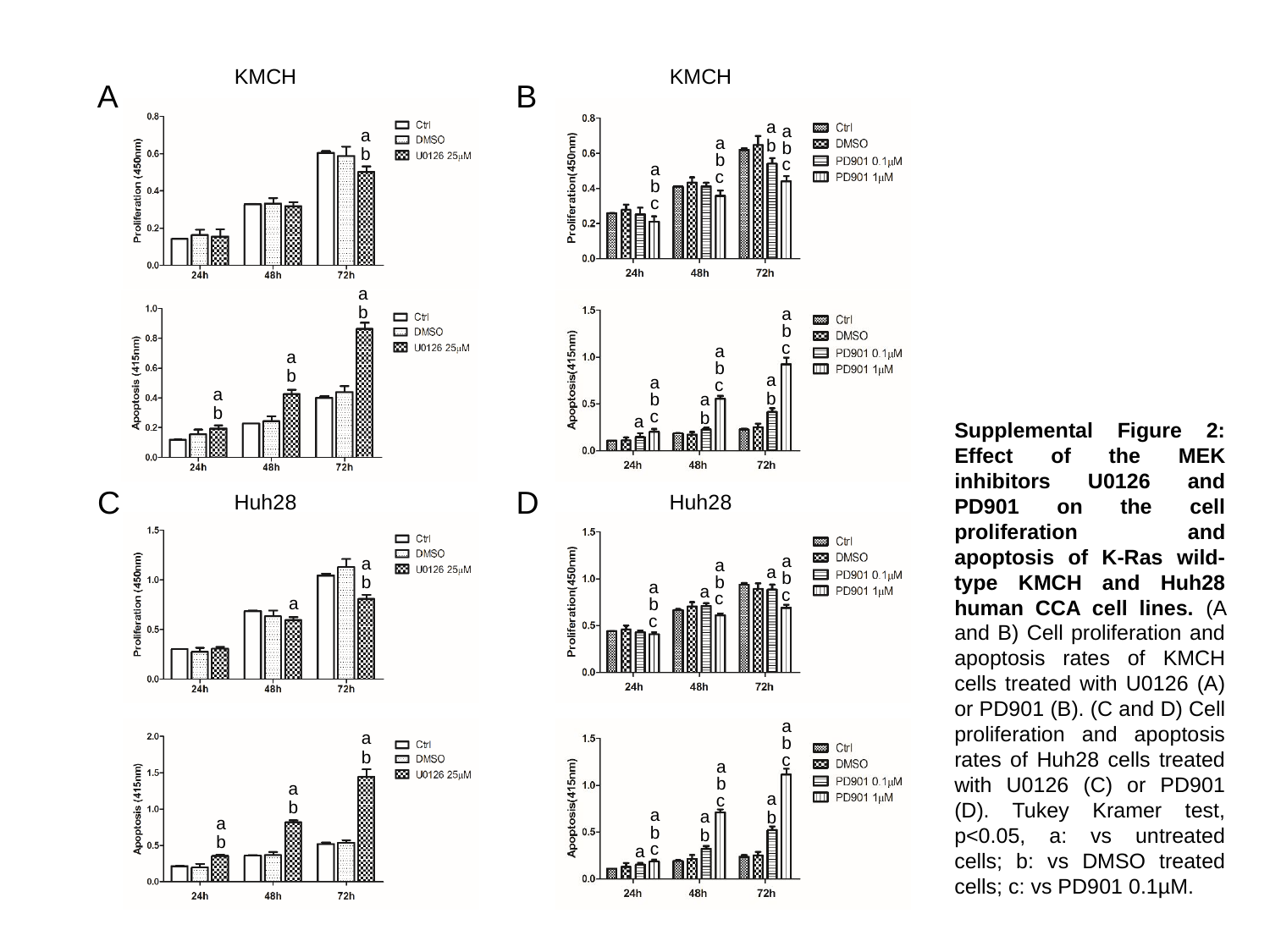

KMCH
KMCH
A
B
a
b
a
b
c
a
b
a
b
c
a
b
c
a
b
a
b
a
b
a
b
c
a
b
c
a
b
a
b
a
b
c
a
b
a
b
a
Supplemental Figure 2: Effect of the MEK inhibitors U0126 and PD901 on the cell proliferation and apoptosis of K-Ras wild-type KMCH and Huh28 human CCA cell lines. (A and B) Cell proliferation and apoptosis rates of KMCH cells treated with U0126 (A) or PD901 (B). (C and D) Cell proliferation and apoptosis rates of Huh28 cells treated with U0126 (C) or PD901 (D). Tukey Kramer test, p<0.05, a: vs untreated cells; b: vs DMSO treated cells; c: vs PD901 0.1µM.
C
D
Huh28
Huh28
a
b
c
a
b
a
b
c
a
a
b
c
a
a
a
b
c
a
b
a
b
c
a
b
a
b
a
b
c
a
b
a
b
a

## Slide 3
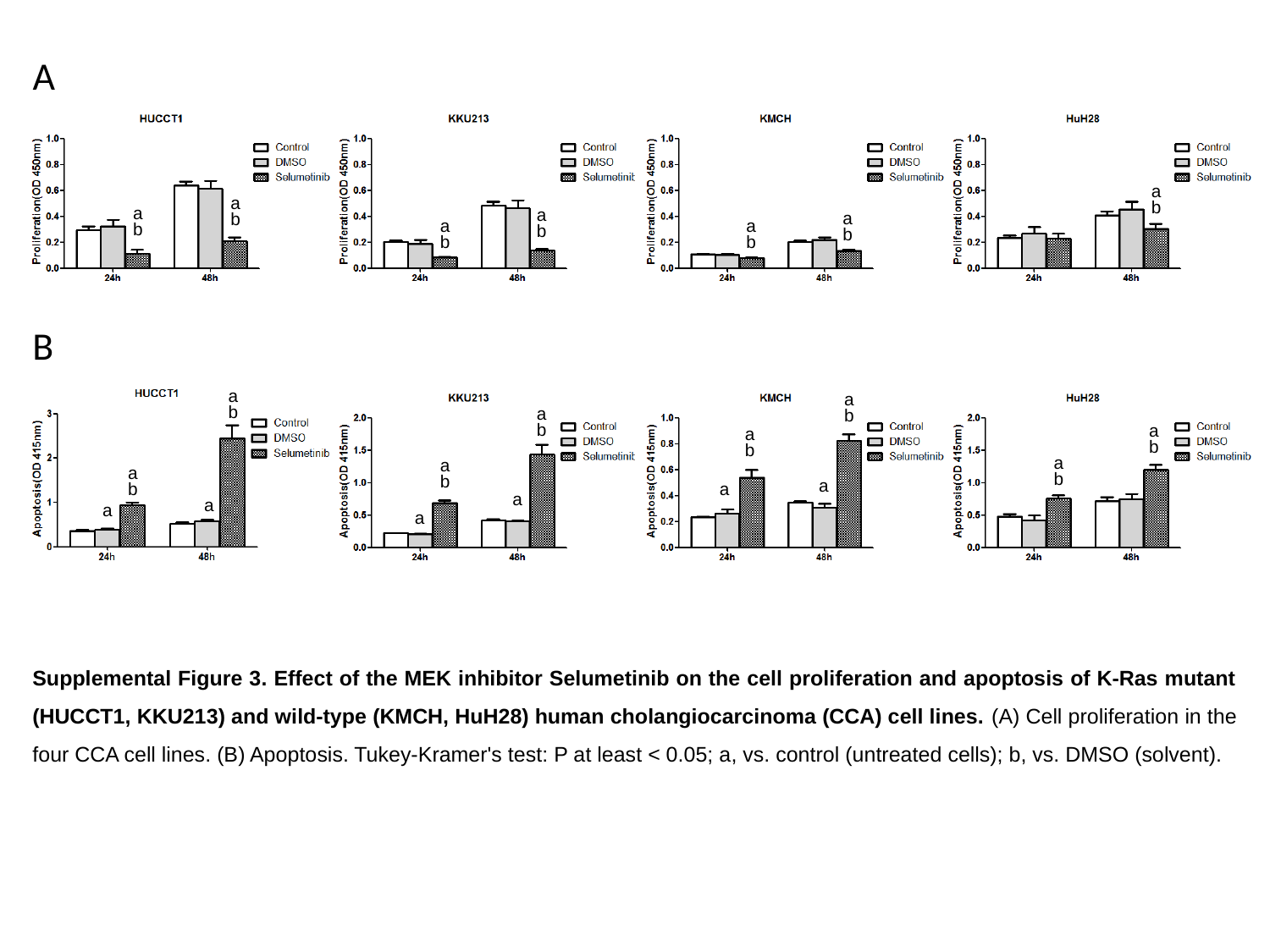

A
a
b
a
b
a
b
a
b
a
b
a
b
a
b
B
a
b
a
b
a
b
a
b
a
b
a
b
a
b
a
b
a
a
a
a
a
a
Supplemental Figure 3. Effect of the MEK inhibitor Selumetinib on the cell proliferation and apoptosis of K-Ras mutant (HUCCT1, KKU213) and wild-type (KMCH, HuH28) human cholangiocarcinoma (CCA) cell lines. (A) Cell proliferation in the four CCA cell lines. (B) Apoptosis. Tukey-Kramer's test: P at least < 0.05; a, vs. control (untreated cells); b, vs. DMSO (solvent).

## Slide 4
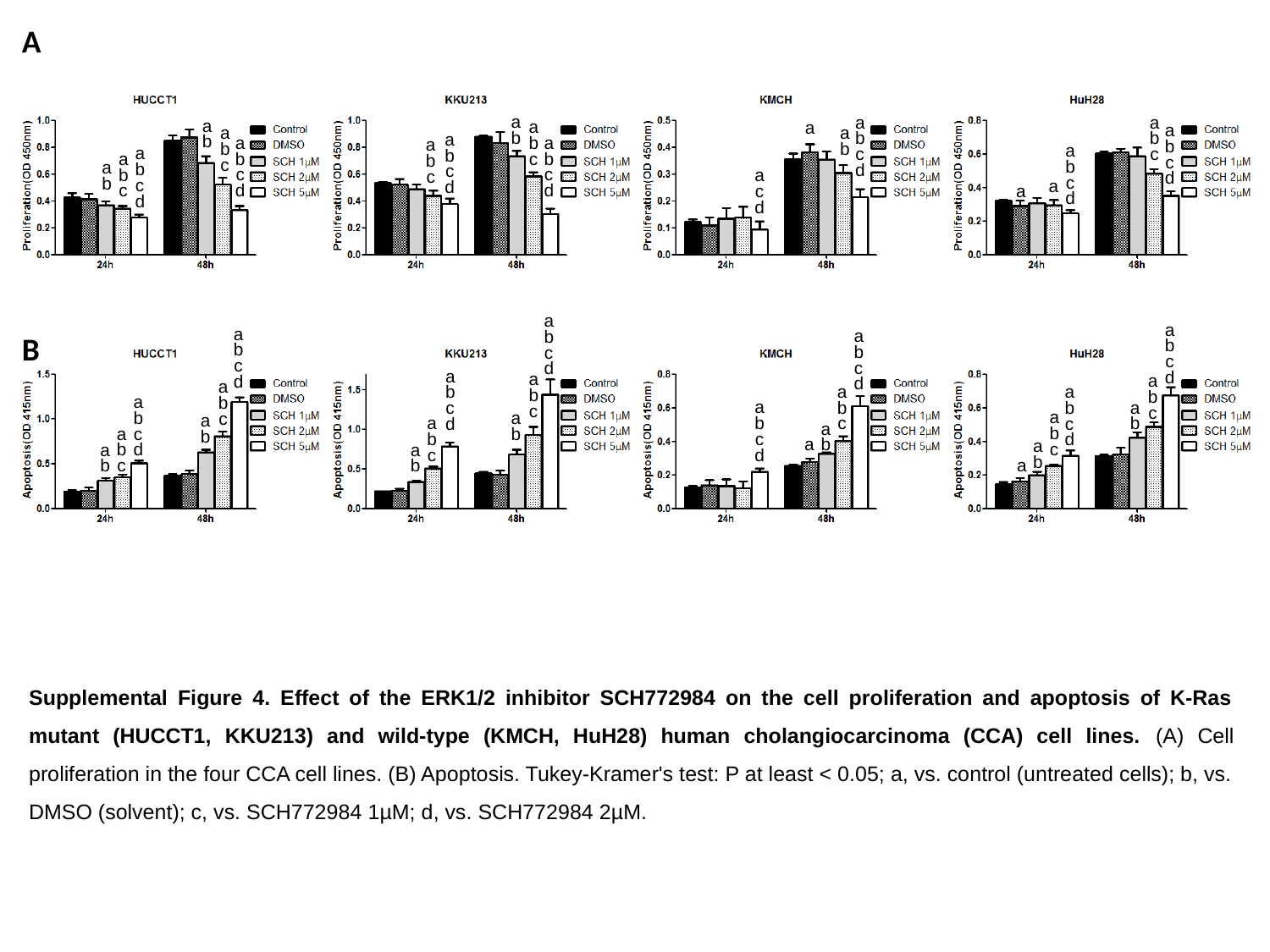

A
a
b
a
b
c
d
a
b
c
a
b
a
b
c
a
a
b
c
d
a
b
c
a
b
a
b
c
d
a
b
c
d
a
b
c
d
a
b
c
a
b
c
d
a
b
c
d
a
b
c
a
b
a
c
d
a
a
a
b
c
d
a
b
c
d
B
a
b
c
d
a
b
c
d
a
b
c
d
a
b
c
a
b
c
a
b
c
a
b
c
a
b
c
d
a
b
c
d
a
b
c
d
a
b
a
b
c
a
b
a
b
a
b
c
a
b
a
b
c
a
a
b
a
b
a
b
a
Supplemental Figure 4. Effect of the ERK1/2 inhibitor SCH772984 on the cell proliferation and apoptosis of K-Ras mutant (HUCCT1, KKU213) and wild-type (KMCH, HuH28) human cholangiocarcinoma (CCA) cell lines. (A) Cell proliferation in the four CCA cell lines. (B) Apoptosis. Tukey-Kramer's test: P at least < 0.05; a, vs. control (untreated cells); b, vs. DMSO (solvent); c, vs. SCH772984 1µM; d, vs. SCH772984 2µM.
