# Supplementary material for: Efficacy of MEK inhibition in a K-Ras-driven cholangiocarcinoma preclinical model
Source: Cell Death Dis. 2018 Jan 18;9(2):31. doi: 10.1038/s41419-017-0183-4 (PMC5833851; doi:10.1038/s41419-017-0183-4)
Supplement: Supplementary file 2 — Supplemental Table 1 2 3 and 4 [file 41419_2017_183_MOESM2_ESM.doc]

**Supplemental Table 1: *K-Ras* mutation status and IC_50_ against U0126 in a panel of human CCA cell lines.**

| Cell lines | *K-Ras* status | IC_50_(μM) |
| --- | --- | --- |
| KKU213 | G13C | 36.4 |
| HuCCT1 | G12D | 47.24 |
| RBE | G12V | 21.7 |
| KMCH | WT | 89.29 |
| HuH28 | WT | 95.2 |
| MzCha-1 | WT | 92.04 |
| OCUG | WT | 64.79 |

**WT: Wild-type K-Ras allele**

**Supplemental Table 2: Immunohistochemistry (IHC) and Western blotting (WB) antibody information**

| Antibody | Company | Catalog number | Dilution | Method |
| --- | --- | --- | --- | --- |
| Phospho-ERK | Cell signaling technology | 4370 | 1:1000 | IHC |
| Myc-tag | Maine Medical Center Research Institute | Vli01 | 1:100 | IHC |
| CK19 | Abcam | Ab133496 | 1:700 | IHC |
| Ki67 | Thermo Scientific | MA5-14520 | 1:100 | IHC |
| Smooth Muscle Actin | DAKO | M0851 | 1:200 | IHC |
| Vimentin | Cell signaling technology | 5741 | 1:100 | IHC |
| AKT | Cell signaling technology | 9272 | 1:1000 | WB |
| Phospho-AKT^S473^ | Cell signaling technology | 3787 | 1:1000 | WB |
| Phospho-AKT^T308^ | Cell signaling technology | 13038 | 1:1000 | WB |
| Phospho-mTOR | Cell signaling technology | 2971 | 1:1000 | WB |
| Myc-tag | Maine Medical Center Research Institute | Vli01 | 1:100 | WB |
| ERK | Cell signaling technology | 9102 | 1:1000 | WB |
| Phospho-ERK | Cell signaling technology | 4370 | 1:1000 | WB |
| Phospho-PRAS40 | Cell signaling technology | 2997 | 1:1000 | WB |
| Phospho-RPS6 | Cell signaling technology | 4858 | 1:2000 | WB |
| Phospho-4E-BP1 | Cell signaling technology | 2855 | 1:2000 | WB |
| Phospho-elF4E | Cell signaling technology | 9741 | 1:1000 | WB |
| PKM2 | Cell signaling technology | 4053 | 1:1000 | WB |
| Survivin | Cell signaling technology | 2808 | 1:1000 | WB |
| PCNA | Cell signaling technology | 2586 | 1:2000 | WB |
| Cyclin A | Santa Cruz Biotechnology | SC-751 | 1:200 | WB |
| Cyclin B1 | Santa Cruz Biotechnology | SC-245 | 1:750 | WB |
| Cyclin D1 | Cell signaling technology | 2978 | 1:10000 | WB |
| Cyclin E | Biolegend | 630701 | 1:200 | WB |
| Cleaved-caspase3 | Cell signaling technology | 9664 | 1:750 | WB |
| Cleaved-caspase7 | Cell signaling technology | 8438 | 1:750 | WB |
| Bcl-2 | Cell signaling technology | 3498 | 1:1000 | WB |
| Bcl-xl | Cell signaling technology | 2764 | 1:1000 | WB |
| Bim | Cell signaling technology | 2933 | 1:1000 | WB |
| Mcl-1 | Cell signaling technology | 94296 | 1:1000 | WB |
| LC-3 | Cell signaling technology | 12741 | 1:1000 | WB |
| β-ACTIN | Sigma-Aldrich | A5441 | 1:4500 | WB |
| GAPDH | EMD Millpore | MAB374 | 1:10000 | WB |

**Supplemental Table 3. Clinicopathological features of intrahepatic cholangiocarcinoma (ICC) patients**

| Variables | | | |
| --- | --- | --- | --- |
| No. of patients  Male  Female | 98  63  35 | |  |
| Age (years)  <60  >60 | 44  54 | |  |
| Etiology  HBV  HCV  Hepatolithiasis  PSC  NA | 10  14  22  3  49 | |  |
| Liver cirrhosis  Yes  No | 21  77 | |  |
| Tumor differentiation  Well  Moderately  Poorly | 37  34  27 | |  |
| Tumor size (cm)  <5 56  >5 42 | |  | |
| Tumor number  Single 62  Multiple 35 | |  | |
| Lymph node metastases  Yes 15  No 47  NA 36 | |  | |

Abbreviations: NA, not available; PSC, primary sclerosing cholangitis

**Supplemental Table 4: Cell lines information**

| Cell lines | Citation | Supplier |
| --- | --- | --- |
| KKU213 | PubMed: 27461717 | JCRB Cell Bank |
| HuCCT1 | PubMed: 2544546 | From Dr. Greg Gores, Mayo Clinics |
| RBE | PubMed: 15767549 | Riken Cell Bank |
| KMCH | PubMed: 3032760 | From Dr. Greg Gores, Mayo Clinics |
| HuH28 | PubMed: 2852388 | JCRB Cell Bank |
| MzCha-1 | PubMed: 4056357 | From Dr. Greg Gores, Mayo Clinics |
| OCUG | PubMed: 1533512 | JCRB Cell Bank |
